# Enhanced Vertical Navigation Using Barometric Measurements

**DOI:** 10.3390/s22239263

**Published:** 2022-11-28

**Authors:** Shrivathsan Narayanan, Okuary Osechas

**Affiliations:** German Aerospace Center (DLR), Münchener Str. 20, 82234 Weßling, Germany

**Keywords:** aircraft navigation, barometric altitude, GNSS altitude, numerical weather model, vertical required navigation performance (RNAV)/(RNP), aircraft vertical guidance

## Abstract

This paper introduces a technique to transform between geometric and barometric estimates of altitude and vice-versa. Leveraging forecast numerical weather models, the method is unbiased and has a vertical error with a standard deviation of around 30 m (100 ft), regardless of aircraft altitude, which makes it significantly more precise than established comparable conversion functions. This result may find application in various domains of civil aviation, including vertical RNP, systemized airspace, and automatic landing systems.

## 1. Introduction

With increasing air traffic, there is a great need to develop systems that enable robust, reliable, and predictable 3-D aircraft navigation, i.e., in the lateral as well as in the vertical domain. At present, reliable navigation services for aircraft can be provided in the lateral domain, i.e., in latitude and longitude. At present, there are clear rules for route/procedural separation in the lateral dimension but the uncertainty around the vertical dimension results in the reservation of large blocks of airspace per procedure. The relatively lower accuracy of vertical is insufficient to provide seamless 3D navigation to service a larger number of aircraft within a given service volume. This necessitates the development of robust and accurate systems that can provide seamless and reliable estimates of aircraft altitude for all phases of flight.

In air-traffic management, two different methods of representing vertical information are widely used: barometric and geometric. Altitude measurements from a barometer also known as pressure altitude. Barometric altitude relies on converting the pressure reading taken at a particular location and converting it to the height above mean sea level using the reference pressure and temperature values provided by the ICAO Standard Atmosphere (ISA) [1]. The conversion allows aircraft to follow isobaric surfaces, expressed in terms that are human-readable in units of feet. It also allows aircraft to follow pre-defined descent profiles. During the approach phase of the aircraft, the reference setting of the barometric altitude is changed from the ISA to the aerodrome depending on whether the aircraft is in cruising altitude or in approach [2,3]. In contrast, the geometric altitude is primarily measured with sensors like Global Navigation Satellite Systems (GNSS) or radar altimeters where the altitude is referenced to either the WGS84 ellipsoid or the local terrain, respectively.

In civil aviation, vertical navigation and localization are typically performed either in terms of a geometric path, or else as a barometric reading expressed, not in units of pressure, but in units of height. The conversion from barometric pressure to height is regulated by the ICAO Standard Atmosphere (ISA) [1] or based on the pressure and temperature measurements from the aerodrome depending on whether the aircraft is in cruising altitude or in approach [2,3]. The conversion allows aircraft to follow isobaric surfaces, expressed in terms that are human-readable in units of feet; it also allows aircraft to follow pre-defined descent profiles.

Controlling aircraft vertical dynamics by barometric measurements has certain advantages dating back to the times before GNSS was pervasive. In the upper airspace, barometric altitude allows for the strategic deconfliction of air traffic going in different directions. Roughly speaking, aircraft going in one direction stick to one isobaric surface, while aircraft going in the opposite direction must be a thousand feet above or below.

The main disadvantage is that barometric measurements of altitude vary with the weather. The ISA does account for that fact but leaves residual biases that can be significant, relative to the requirements associated with the vertical guidance in landing the aircraft. Using the ISA as a measurement of altitude can lead to errors over several hundred feet. The current methodology as described in the DO-236C/ED-75D [2,3], cannot distinguish between pressure changes that occur due to changes in altitude or due to the passage of weather fronts. This is, however, not an issue for conventional operations, as these errors are either common for all aircraft in an area, thus maintaining vertical separation, or can be compensated by referring locally to a known altitude such as an aerodrome.

By contrast, GNSS-based measurements of height are unbiased and can achieve accuracies in the order of a few meters. The advantage of GNSS-based altitude over barometric altitude is that they are not affected by variations in the atmosphere. With adequate processing, satellite navigation services offer sufficient performance to support all phases of flight, most notably departure, en-route, and approach. Note that satellite services can also support more stringent operations, such as precision approach and landing, a similar analysis of the methodology proposed in this paper is left as an item for future work.

The method presented in this paper: BiG-C (Barometric to Geometric Converter), offers a way of converting barometric estimates of altitude to geometric altitude and vice-versa. The proposed algorithm improves the vertical repeatability of barometric altitude by accounting for the impact of off-nominal atmospheric effects such as weather fronts or ducting on barometric altitude measurement. By using numerical weather forecasts, these conversions have negligibly small biases and a residual uncertainty that is small enough to support navigation services in the vertical for most en-route, as well as departure and arrival applications.

The proposed conversions are interesting, in civil aviation applications, for a variety of reasons. These include improved repeatability in the vertical position, reduced workload in the cockpit, and vertical guidance in case of a GNSS failure, to name a few. An unbiased conversion function between barometric and geometric altitudes, and vice versa, would find wide application in making air traffic management more efficient.

DO-236C [2]/ED-75D [3] describes the Vertical Path Performance Limits (VPPL) that an altitude measurement system must comply with for different phases of flight. The most stringent VPPL value is about ±48.768 m (160 feet) for aircraft altitudes below 5000 feet [2,3]. Thus, in the context of this paper, any residual error below ±24.384 m (80 feet) is considered negligible.

## 2. Materials and Methods

An altimetry system based on barometric measurements must be able to provide real-time, robust, and repeatable altitude estimates, irrespective of any atmospheric variability. The underlying methodology of the BiG-C and the corresponding validation setup described in this paper leverages the forecast NWM to demonstrate a near bias-free conversion between the barometric and geometric altitudes. The evaluation of the BiG-C methodology is performed by validating its behavior with respect to the GNSS altitude collected as part of flight experiments conducted by the German Aerospace Center (DLR).

### 2.1. Operational Background

Different terminologies are utilized to differentiate the altitude derived from barometric measurements based on the reference surface with respect to which the altitude is derived. The reference surface selected depends on the barometric pressure set on the altimeter sub-scale. Three references for barometric pressure are in common usage: QNH, QFE, and Flight levels.

- QNH: This refers to the height above the mean sea level (MSL), i.e., the height above the geoid or also referred to as the orthometric height (H). The QNH pressure values correspond to the local sea level pressure (with the ISA lapse rates) provided by the nearest ground stations.
- QFE: This refers to the height above the runway. Here, the local measurements in the airport are transmitted to the aircraft to calibrate the barometric altimeter. In the PANS-OPS Doc 8400 [4], see Q-Codes, QFE is referred to as “Atmospheric pressure at aerodrome elevation (or at runway threshold)”. With the aerodrome QFE set in the subscale, your altimeter will read zero on the highest point on the runway, and at other altitudes will read the height above the airfield elevation.
- Flight Levels: Flight levels are used to ensure safe vertical separation between the aircraft, despite local fluctuations in atmospheric pressure and temperature. It is used by all aircraft operating above the transition altitude to provide a common reference for vertical measurement. Though this setting is used to maintain the aircraft separation, it does not give the actual height of the aircraft above sea level or the ground. Thus, we use the transition altitude to indicate the shift from calibrating the barometer to QFH to using flight levels. While operating at or below the transition altitude, the aircraft altimeter shows the altitude above the local runway. Additionally, when above the transition altitude, the barometer is adjusted to the ISA pressure surface (i.e., with respect to 1013.25 hPa).

Each aircraft operating in airspace where vertical performance is specified shall have total system error components in the vertical direction that are less than the specified performance limit 99.7% of the flying time [2,3]. To ensure that the airplane remains on the desired path, certain limits are established for errors in navigation performance. Table 1 illustrates the vertical path performance limit (VPPL) of the navigation system, including static source pressure altimetry error as stated in DO-236C [2]/ED-75D [3].

**Figure 1:**
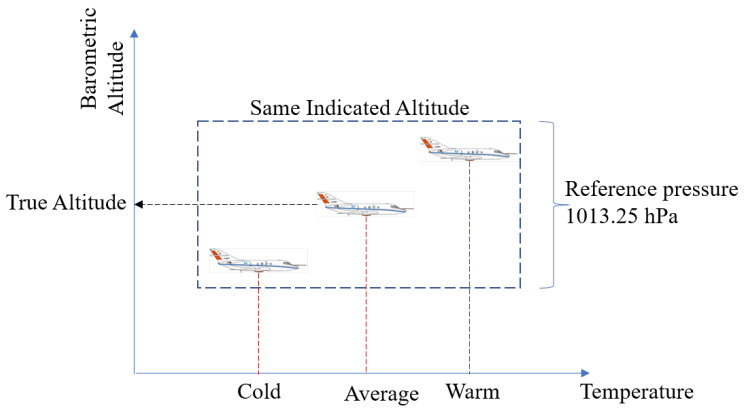
The indicated altitude significantly deviates from the true altitude of the aircraft based on the variation of the temperature from the ISA reference values in warmer or colder weather.

The assumption of ICAO standard atmospheric (ISA) values results in significant deviations in the derived altitude between warmer or colder climates. The assumption of an ISA would be acceptable at high altitudes since all the aircraft make similar assumptions and are all making the same error in their vertical altitude. Since at these altitudes the important thing is to maintain the required minimum separation between the aircraft. However, during the approach phase of the aircraft, the assumption of the standard ISA atmosphere is not sufficiently accurate due to the following reasons:

- If the temperature at the sea level is different from 15 C then the rate of decrease in pressure will be different and thus will lead to erroneous altitude measurements. For example, as shown in Figure 1, on a warm day the altimeter will yield a higher altitude, and on a cold day, the altimeter will under-read, for the same pressure measurement from the barometer.
- The surface pressure varies significantly when compared to the ISA or the QNH measurement.

The current solution specified in DO-236C/ED-75D requires temperature compensation functions to correct for the difference in the temperature between the ISA and the atmosphere in which the aircraft is flying. An inherent problem with the present solution is that even with temperature corrections for the reference level, the present approach of utilizing the ISA does not account for altitude variations of pressure and temperature in real time. Thereby, neglecting any changes in the pressure and temperature profiles and the resulting impact on the barometric altitudes due to severe atmospheric conditions such as weather fronts, ducting, etc. In addition, the current approach of utilizing the temperature compensation functions is only applicable below the transition altitude and when the aircraft is in the vicinity of the airport. Thus, above the transition altitude, the barometric altitudes still exhibit significantly large altitude differences resulting from the deviation of the ISA from the true state of the atmosphere.

In addition to the correction functions to compensate for atmospheric variability, the pilot has to manually set the reference level to QNE or QFE for the barometric altitude measurements depending on its altitude. Occasionally, a pilot may forget to reset the barometric pressure reference setting to standard (1013.25 hPa) when transitioning through the transition altitude. This can lead to the aircraft flying at a different altitude than that assigned. Aircraft cruise within flight routes at assigned altitudes. If an aircraft is not flying at its assigned altitude, it becomes a threat to other aircraft operating in the same flight route.

Once the aircraft is above the transition altitude the barometric setting is changed to QNE to maintain the altitude measurements with reference to the MSL using the ISA reference values for pressure and temperature. Once the aircraft is below the transition altitude the pilot needs to manually change the barometric reference to QFE where the reference values are utilized as measured from the specific airfield. Non-uniform transition altitudes defined across the world (varying significantly between 18000 feet in the USA to various altitudes based on the location of the airport), lead to a significant pilot workload to ensure an accurate reference plane is chosen along with the appropriate corrections being applied to compensate any deviation from the ISA.

### 2.2. Theoretical Background

Altimeter measurements are relative to the reference surface, which could range from sea level to the local terrain (such as the mountain top). If airborne data are to be compared, it is necessary to introduce a reference surface to which all measurements are compared. Historically, the mean sea level (MSL) has been used as the zero of elevation, and this is closely approximated by an equipotential surface for the earth’s gravity field called the geoid [5]. Although much smoother than the topographic surface of the earth, the geoid has significant vertical undulations due to the large-scale distribution of the earth’s mass. A much smoother reference surface is the reference ellipsoid, which is used to approximate the shape of the earth. The reference ellipsoid is convenient because three-dimensional coordinates (latitude, longitude, and altitude) are easily defined with respect to it.

Definitions of height and altitude vary depending on the vertical datum or reference level used. In dealing with airborne research data, it is common to encounter different technical terms relating to altitude and vertical direction. These include geometric altitude, GNSS altitude, pressure altitude, and geopotential height. Two altitude scales are widely used in Air Traffic Management (ATM): geometric altitude and geopotential altitude or height. Geometric altitude is the scale that is typically referenced to the WGS-84 ellipsoid or the local terrain. On the other hand, the geopotential height is referenced to the so-called geoid or the mean sea level (MSL) [5].

**Figure 2:**
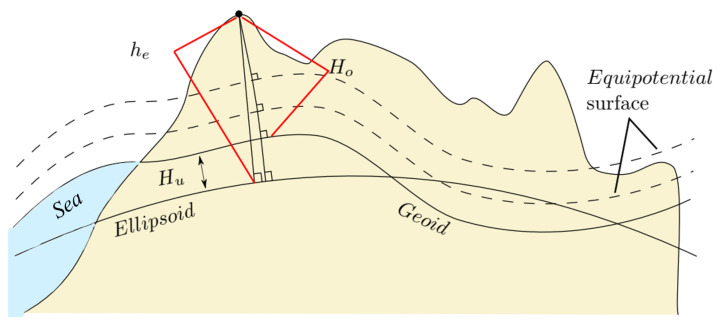
Different vertical data (geoid or ellipsoid) are possible depending on the selected approximation for the earth’s surface. The corresponding heights for the respective vertical data are ellipsoidal height ($h_{e}$), and orthometric height ($H_{o}$). These two height systems are related by the geoid height $H_{u}$.

Figure 2 illustrates the relationship between various surfaces that are used as a reference for measuring height in individual height systems. As seen in Figure 2, the most common vertical datums used in geodesy and navigation applications are [5]:

- **Ellipsoidal Height**: The normal distance of a point from the reference ellipsoid to a point above the earth’s surface is termed the ellipsoid height ($h_{e}$). The most recent version of the reference ellipsoid is the WGS-84, which is also used by the Global Position Systems (GPS).
- **Orthometric Altitude**: The height normal to the geoid is called the orthometric height ($H_{o}$). In geodetic terminology, the term geometric altitude specifies the reference as mean sea level, which means the perpendicular measured distance is related to the geoid. Thus, the geometric height of a point relative to a geoid is usually related to the orthometric height.
- **Geoid Undulation or Geoid Height**: The normal distance, which can be positive or negative, from the reference ellipsoid to the geoid is called geoid height/undulation ($H_{u}$). The geoid undulation is dependent on latitude and longitude. Different values of gravity can be used to determine $H_{u}$. Nivenski [6] compared different gravity values such as normal gravity, normal gravity without centrifugal contribution, and an EGM96 expansion of the actual gravity, and found that the impact of using different gravity formulas on the geoid undulation calculation is insignificant. In this work, we use the geoid undulations defined by the EGM-96 earth gravitational model.

**Figure 3:**
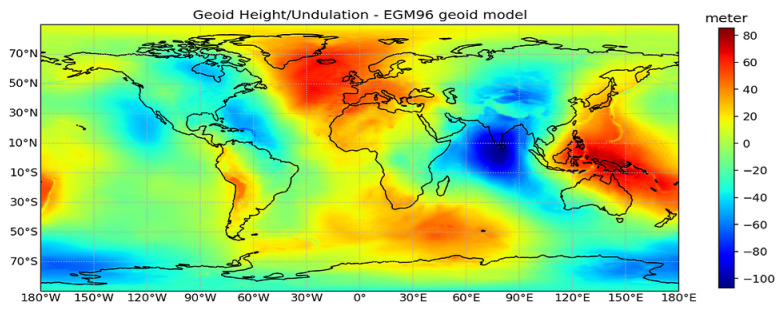
Selecting an appropriate vertical datum for height measurements at or above the earth’s surface is crucial, as the Geoid (mean sea level) and the WGS84 reference ellipsoid can vary by as much as ±100 m.

The height at or above the earth’s surface can be measured with respect to the reference ellipsoid or the geoid, and these can differ by as much as ±100 m. As described before the geoid undulation ($H_{u}$) is required for the transformation between both reference systems. Figure 3 shows the geographic variability of the geoid undulations (i.e., the difference between the approximation made by the reference ellipsoid of the earth’s equipotential surface and the mean sea level). At each latitude and longitude, the height of the geoid above the WGS84 ellipsoid is computed using interpolation of a grid of values for the EGM-96 earth gravity model. For this cubic interpolation is used. The RMS error of using the interpolated height is about 1 mm [7]. The errors in the calculation of geoid undulation values primarily stem from (1) the method of interpolation, (2) the height ambiguity resulting from the fact that the geoid and ellipsoid are not parallel surfaces, and (3) the grid format of the geoid harmonic coefficients, which require quantization of height to 3 mm [7]. We observe that the geoid undulations are not negligible in most continental regions, with significant variability ranging from −100 meters (in the Indian subcontinent) to around +80 m in Europe.

Geopotential altitude (ζ) is referenced with respect to the mean sea level (MSL), at a constant gravity acceleration at 45 latitude. Radiosondes and NWM data generally report geopotential height—a scale that relates height to gravitational equipotentials, or surfaces of constant gravitational potential energy per unit mass. Thus, the geopotential height is constant if one follows the same gravity potential, as one moves from place to place. Although geopotential height approximates geometric height, they are not the same. An important type of geopotential height is pressure altitude, which is based on a standard atmospheric model for temperature as a function of pressure. One particular model, the ICAO ISA [1], is what all aircraft altimeters use to relate static pressure measurements on an aircraft to a corresponding pressure altitude scale.

The gravitational potential energy or geopotential (Φ) of a unit mass of anything is simply the integral from mean sea level (*z* = 0 m) to the height of the mass (*z* = *h*), given as [5]:

(1) $$\Phi =\int_{0}^{h}g\left(\phi ,z\right)\;dz\;\left[\frac{m^{2}}{s^{2}}\right]$$

where, g(ϕ,z) is the gravity acceleration above the geoid at geodetic latitude ϕ and geometric altitude *z*. Now, while the geopotential (potential energy per unit mass) is useful for atmospheric dynamics studies (since it is a convenient way to compare meteorological data from different locations), it is more convenient if it is expressed as a height above the geoid. Thus, the geopotential (Φ) is divided by the normal gravity ($g_{45}$) at a latitude of 45 degrees to obtain the geopotential height as [5]:

(2) $$\zeta =\frac{\Phi }{g_{45}}\;\left[m\right].$$

**Figure 4:**
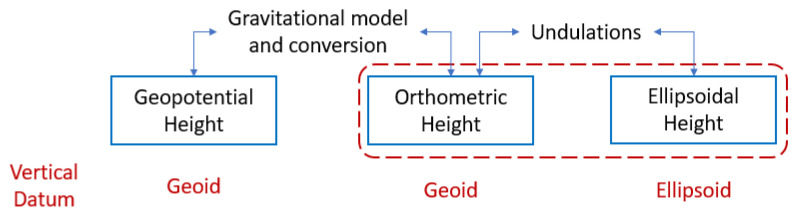
The transformation between geopotential altitude and ellipsoidal height is a non-linear process and depends on the underlying expressions used for the surface gravity on an ellipsoid and geoid undulations (which in turn depends on the geoid model).

Transformation of geopotential to orthometric height is a non-linear process. As described in Figure 4 and in Equations (1) and (2), we need to account for the variation of gravity with altitude. In general, this is handled by the means of encoding the variation of gravity with latitude and height using a fictitious, latitudinally varying, “effective earth’s radius” ($R_{eff}$) [5], given as:

(3) $$R_{eff}\left(\phi \right)=\frac{a}{1+f+m-2f\sin^{2}\left(\phi \right)}.$$

Following this, the geopotential height can be transformed into orthometric (geometric) height as [5]:

(4) $$H_{o}=\frac{R(\phi )\cdot \zeta }{\frac{g(\phi ,H_{o})}{g_{45}}\cdot R\left(\phi \right)-\zeta }$$

where, $g_{s}\left(\phi ,H_{o}\right)$ is the gravity acceleration at the latitude ϕ and at height $H_{o}$. Note that estimating the orthometric height using Equation (4), in turn, depends on the value of gravity at the given orthometric height ($H_{o}$). This leads to an iterative and non-linear process.

Now, for civil aviation, the atmospheric effects need to be accounted for up to the mesosphere (altitude of ∼80 km), which is considerably small in comparison to the earth’s radius. Within the mesosphere, the altitude variability of the gravity over the ellipsoid is found to be negligibly small and the fractional difference in the geopotential height is primarily dominated by the variation in surface gravity [8]. Therefore, it is sufficient to evaluate the gravity (described in Equation (4)) on the surface of the ellipsoid. The resulting expression for the orthometric height is given as [5],

(5) $$H_{o}=\frac{R(\phi )\cdot \zeta }{\frac{g_{s}\left(\phi \right)}{g_{45}}\cdot R\left(\phi \right)-\zeta }$$

where, $g_{s}\left(\phi \right)$ is the normal gravity on the surface of an ellipsoid of revolution for a given latitude (ϕ), which is based on the Somigliana’s expression [5], given as:

(6) $$g_{s}\left(\phi \right)=g_{0}\left(\frac{1+k\sin{\left(\phi \right)}^{2}}{\sqrt{1-e^{2}\sin{\left(\phi \right)}^{2}}}\right)$$

where, *k* is the Somigliana’s constant [5]. Note that the resulting difference in the orthometric height due to the simplification introduced in Equation (5) is negligibly small within mesosphere [8].

Finally, the orthometric height ($H_{o}$) is transformed into ellipsoidal height ($h_{e}$) using the geoid undulation ($H_{u}$) as,

(7) $$h_{e}=H_{o}\pm H_{u}.$$

In controlled airspace, aircraft are typically assigned flight levels, which are defined in terms of pressure but expressed in units of altitude (i.e., feet). Atmospheric pressure varies in space and time; if an aircraft were to maintain a constant geometric altitude, the barometer would read different pressure values. Thus, to have a constant barometric altitude reading, the ISA model must be updated with local atmospheric conditions. The ISA is described with a sea level pressure of 1013.25 hPa and temperature of 15 C, and thus may not correspond to the aircraft’s actual altitude above the true mean sea level or above the ground level.

### 2.3. Computational Setup: Improved Altitude Measurements from Barometric Data

**Figure 5:**
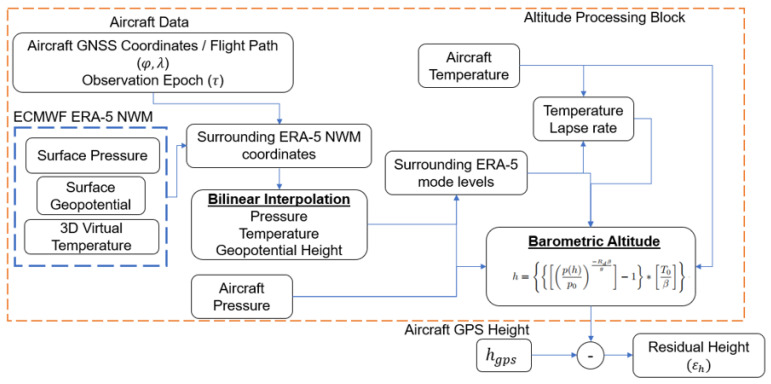
Computational setup for estimating barometric altitude.

To validate the proposed methodology the computational setup compares the aircraft altitude in two ways: from GNSS altitude derived from the improved barometric altitude (h) and from the GNSS altitude ($h_{gps}$) available from the GNSS receiver onboard the aircraft. Figure 5 describes the various steps involved in estimating and validating the improved barometric altitudes from the NWM with the GNSS altitude.

The numerical data underlying the ray tracing stems from the ERA-5 European Center for Mid-Range Weather Forecasts (ECMWF) forecast and re-analyses model level numerical weather model (NWM). This comprises the required meteorological data: surface geopotential and pressure, together with 3D temperature on 60 model levels, at a horizontal resolution of 0.125 × 0.125 degrees in latitude and longitude, and sampled every 6 h.

The meteorological data from NWM are available in a 3D field of latitude ϕ, longitude λ, and height *h* with a certain horizontal and vertical resolution. The finest or highest horizontal resolution is 0.125 × 0.125in latitude and longitude [9]. Since the NWM is the source of the meteorological data to generate the atmospheric reference values for the barometric altitude computation, its horizontal resolution is set to that of the NWM.

In meteorology, the virtual temperature is often used instead of the static temperature [10]. Compared to the temperature of a moist air mass, this is the theoretical (higher) temperature that an equivalent dry air parcel with the same properties would have, such as density and pressure. The use of this abstract variable simplifies the calculations within NWP models because it considers the moisture content of the air while allowing the use of mathematical expressions that are normally only used for dry air. Thus, we must use virtual temperature in the computation of the reference NWM level temperature that will be used in the computation of barometric altitude. The virtual temperature is computed as [10],

(8) $$T_{v}=T\left(1+\left[\frac{R_{d}}{R_{w}}-1\right]\cdot q\right)$$

where *T* is temperature, *q* is specific humidity and $R_{d}$ and $R_{w}$ are the universal gas constants for dry and moist air, respectively.

**Figure 6:**
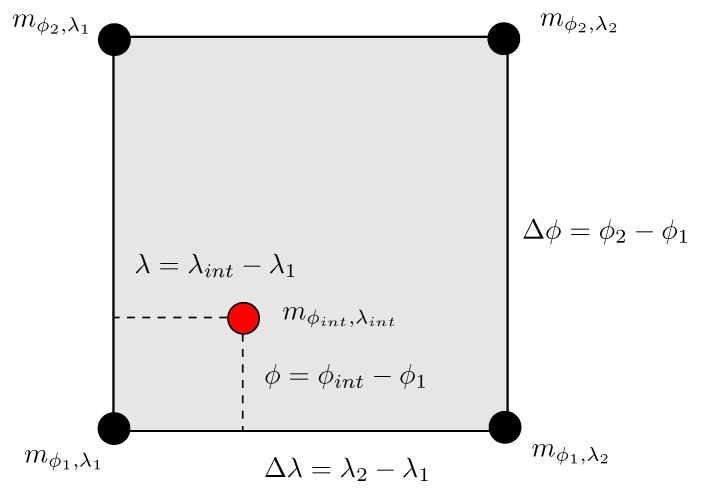
Geometry for bilinear interpolation of the refractive index at aircraft coordinates $m_{\phi_{int},\lambda_{int}}$ using the surrounding refractive indices $m_{\phi_{1},\lambda_{1}}$, $m_{\phi_{1},\lambda_{2}}$, $m_{\phi_{2},\lambda_{1}}$, $m_{\phi_{2},\lambda_{2}}$.

Most often the location of the aircraft does not coincide with the coordinates where the NWM is available. Therefore, for a given aircraft coordinate (latitude and longitude), we horizontally interpolate the NWM data at all model levels, that spatially surround the aircraft location. This provides a 3D snapshot of the meteorological parameters at the exact aircraft latitude and longitude. In this work, we use bilinear interpolation (as shown in Figure 6) of the constituent parameters such as pressure, geopotential, and virtual temperature to compute the corresponding parameters at all model levels coinciding with the latitude and longitude of the aircraft. The use of bilinear interpolation for the meteorological parameter estimation is a well-tested and commonly used technique in GNSS and atmospheric propagation delay modeling [11].

Figure 6 illustrates the bilinear interpolation of the NWM data from a set of surrounding grid points. As seen in the figure, considering a 2D grid of nodes, the NWM data (pressure, temperature, and specific humidity) at the desired location denoted as $m_{\phi_{int},\lambda_{int}}$ is obtained by considering the data $m_{\phi_{1},\lambda_{1}}$, $m_{\phi_{1},\lambda_{2}}$, $m_{\phi_{2},\lambda_{1}}$ and $m_{\phi_{2},\lambda_{2}}$ given at the grid nodes: $\left(\phi_{1},\lambda_{1}\right)$, $\left(\phi_{1},\lambda_{2}\right)$, $\left(\phi_{2},\lambda_{1}\right)$ and $\left(\phi_{2},\lambda_{2}\right)$ that surround the desired location $\left(\phi_{int},\lambda_{int}\right)$. The surrounding nodes must satisfy the following conditions:

(9) $$\begin{array}{cccc} \phi_{1}\leq \phi_{int} & \leq \phi_{2} & \;\;\;\;\lambda_{1} & \leq \lambda_{int}\leq \lambda_{2} \end{array}.$$

The meteorological data $m_{\phi_{int},\lambda_{int}}$ at the aircraft coordinates $m_{\phi_{int},\lambda_{int}}$ is evaluated as:

(10) $$m_{\phi_{int},\lambda_{int}}=\left(1-\chi \right)\left(1-\xi \right)m_{\phi_{1},\lambda_{1}}+\left(1-\chi \right)\xi m_{\phi_{1},\lambda_{2}}+\chi \xi m_{\phi_{2},\lambda_{2}}+\chi \left(1-\xi \right)m_{\phi_{2},\lambda_{1}}$$

where χ and ξ denote weighting parameters for the contribution of each grid node value to the interpolation result. They are given as:

(11) $$\begin{array}{cccc} \chi & =\frac{\phi_{int}-\phi_{1}}{\phi_{2}-\phi_{1}} & \;\;\;\;\xi & =\frac{\lambda_{int}-\lambda_{1}}{\lambda_{2}-\lambda_{1}} \end{array}.$$

Most often the epochs at which the NWM is available do not match the time at which the flight data is collected, thus, we interpolate the atmospheric parameters to the exact observation times. The atmospheric parameters retrieved from the NWM are only valid at a specific time with respect to the time at which the NWM is available. The ERA-5 NWM is available with a time resolution of one hour. This fundamental time resolution of the NWM is sufficient to compute the atmospheric variables using the two epochs that temporally surround the desired time of observation at which we need to compute the barometric altitude. Thus, in this case, a linear interpolation between the NWM determined at the surrounding epochs leads to the final geopotential, pressure, and virtual temperature at the exact observation time. Reference [12] describes the linear interpolation of tropospheric delays with respect to the time domain,

(12) $$m_{t_{obs}}=m_{t_{ep1}}+\frac{m_{t_{ep2}}-m_{t_{ep1}}}{t_{ep2}-t_{ep1}}\left(t_{obs}-t_{ep1}\right)$$

where $m_{t_{obs}}$ is the interpolated meteorological data at the observation time $t_{obs}$. $t_{ep1}$ and $t_{ep2}$ are the NWM epochs at which the NWM data are available. Since the meteorological data at $t_{obs}$ is calculated from the surrounding NWM epochs, $t_{ep1}$ and $t_{ep2}$ additionally fulfill the following condition:

(13) $$t_{ep1}\leq t_{obs}\leq t_{ep2}.$$

The conventional exponential distribution used to describe the variation of pressure with altitude is not applicable for altitudes with tropopause (∼12 km), which is the usual operational altitude for civil aviation [12]. In contrast, within the tropopause, the temperature variability with altitude is well described by a linear model [13],

(14) $$T=T_{0}-\beta \left(h-h_{0}\right)$$

where, $T_{0}$, β, and $h_{0}$ are the reference temperature, altitude, and temperature lapse rate, respectively. Using this expression for temperature in the equation of hydrostatic equilibrium and integrating from the surface to a height *h*, we obtain the expression for the variation of pressure with altitude as,

(15) $$p\left(h\right)=p_{0}{\left(1-\frac{\beta (h-h_{0})}{T_{0}}\right)}^{\frac{g_{0}}{R_{d}\beta }}.$$

Thus, re-arranging the equation for pressure (15), for a given reference pressure ($p_{0}$) and temperature $T_{0}$, we can compute the altitude (*h*) for a given pressure measurement (p(h)) as,

(16) $$h=\frac{T_{0}}{\beta }\left[1-{\left(\frac{p(h)}{p_{0}}\right)}^{\frac{R_{d}\beta }{g}}\right]+h_{0}.$$

Equation (16) is known as the barometric altitude.

Utilizing a reference pressure and temperature from NWM accounting for the variability in temperature and pressure gradients in the atmospheric column beneath the aircraft yields the most accurate estimates of the barometric altitude. The nominal atmosphere is usually characterized by a temperature lapse rate of 6.5 K/km. However, most often the atmosphere deviates from the nominal temperature profile, especially in the presence of off-nominal atmospheric effects such as ducting [14,15]. Ducting causes the temperature gradient to be either zero or positive, which results in the temperature being either constant or increasing with an increase in altitude [14,15]. As seen in Equations (14) and (15), variations in temperature lapse directly impact the vertical profile of pressure and temperature. This leads to a non-linear vertical temperature gradient. Since the measurement of barometric altitude depends on the temperature lapse rate, deviations in temperature profile from a linear model will result in errors in the estimated altitude. Thus, utilizing the mean sea level pressure and temperature values does not yield the most accurate reference for estimating the barometric altitude.

**Figure 7:**
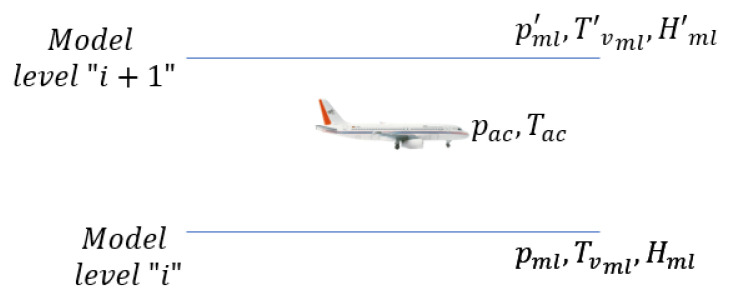
Computation of reference surface values and temperature lapse rate for estimating the barometric altitude from a pressure and temperature measurement at aircraft.

To account for the impact of non-ideal atmospheric variability in the barometric Equation (16), we use the following procedure, as described in Figure 7:

- NWM provides static pressure $p_{ml}$, virtual temperature $T_{v_{ml}}$, and geopotential altitude $H_{ml}$ at discrete altitude levels.
- For a given pressure and temperature measurement at the aircraft altitude, we use the bilinear interpolated NWM data determined at the latitude and longitude of the aircraft and compare the pressure measurement from the aircraft with the pressure data at all model levels from the NWM.
- This yields the model levels with pressure values that surround the aircraft pressure data.
- The surrounding model levels are used as the reference surface to determine the temperature lapse rate,
  (17) $$\beta =\frac{T_{v_{ml}}^{\prime }-T_{v_{ml}}}{h_{ml}^{\prime }-h_{ml}}$$
- Next, using the temperature lapse rate computed based on Equation (17), and using the reference pressure and temperature from the model levels surrounding the aircraft, we compute the aircraft altitude using the barometric equation.
- Next, the geopotential altitude ($H_{ml}$) of the reference model level is added to the derived altitude (*h*) to obtain the barometric altitude of the aircraft above the mean sea level.
- Finally, we validate the barometric altitude obtained from step 6 by comparing it against the GNSS altitude ($h_{gps}$).

Since the pressure and temperature at each model level of the NWM account for the variability in atmospheric parameters in the model levels below it, utilizing a reference surface for the barometric equation that surrounds the aircraft pressure measurement, inherently considers any deviation of the temperature and temperature lapse rate due to off-nominal atmospheric effects such as ducting.

### 2.4. Validation Setup

In real-time application of the proposed methodology, the ERA-5 forecast NWM provides near real-time reference pressure and temperature values to compute barometric altitude. We use the 6-hour and 12-hour forecast of the ERA-5 NWM data from ECMWF to generate the required meteorological information for computing the pressure, temperature, and the associated temperature lapse rates to be used in the barometric equation.

**Figure 8:**
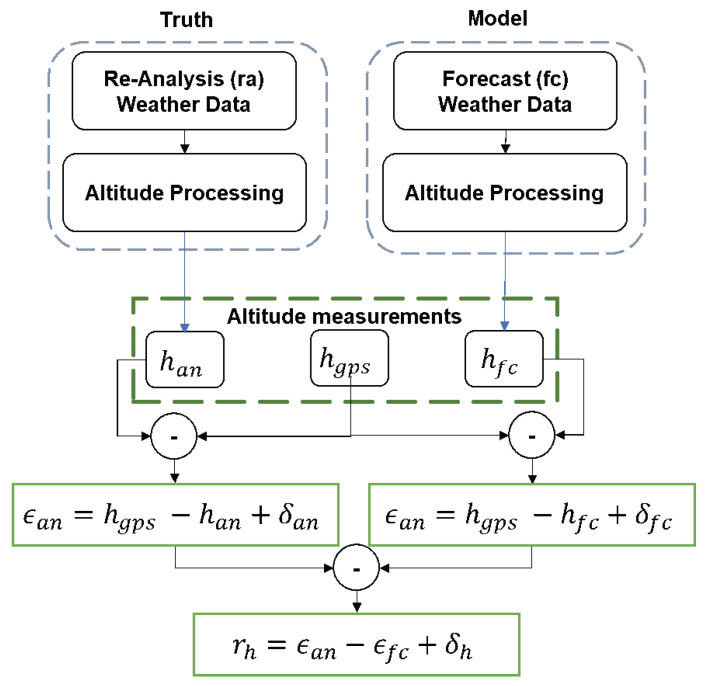
Computational setup for validating the accuracy of barometric altitude corrections using forecast numerical weather data.

To validate the residual error of the proposed altitude estimation methodology, the computational setup compares barometric altitude in two ways: by using the reference pressure and temperature profiles from a more accurate re-analysis of NWM and from the forecast NWM with the GNSS altitude. Figure 8 outlines the flowchart indicating the various steps involved in validating the methodology using the forecast NWM to accurately estimate the barometric altitude.

In general, the accuracy of the proposed methodology to provide the required reference pressure and temperature values to compute the barometric altitude depends on the ability of the forecast NWM to represent the state of the atmosphere. To validate the accuracy of the forecast NWM, we utilize the re-analysis NWM. Reanalysis data provide the most complete picture currently possible of past weather and climate. They are a blend of observations with past short-range weather forecasts rerun with modern weather forecasting models. They are globally complete and consistent in time and are sometimes referred to as ’maps without gaps’. Reanalysis combines past short-range weather forecasts with observations through data assimilation. The process mimics the production of day-to-day weather forecasts, which use an analysis of the current state of the Earth system as their starting point. The analysis is a physically consistent blend of observations with a short-range forecast based on the previous analysis. A detailed analysis of the accuracy of the forecast NWM to predict various atmospheric parameters including off-nominal atmospheric effects is given in [15].

The forecast NWM accurately predicts the atmospheric parameters even in presence of off-nominal atmospheric in comparison to the reference re-analysis NWM. Recent work showed that off-nominal atmospheric tropospheric effects such as ducts are not particularly rare and can result in significant deviations in the pressure and temperature profiles in comparison to the nominal behavior described in Equations (14) and (15) [14,15]. The ability of the forecast NWM to accurately provide the reference pressure and temperature values to estimate the barometric altitude is determined as follows:

- As described earlier, the reference aircraft altitude information is obtained from GNSS ($h_{gps}$).
- The pressure, temperature, and the temperature lapse rate profiles from the forecast and re-analysis NWM are used within the altitude processing block described in Figure 5 to estimate the corresponding barometric altitude $h_{fc}$ and $h_{an}$, respectively.
- The resulting altitude difference using the re-analysis NWM with respect to GNSS altitude is given as:
  (18) $$\epsilon_{an}=h_{gps}-h_{an}+\delta_{an}$$
  where $\delta_{an}$ is the error resulting from the spatial and diurnal interpolation of the re-analysis of NWM data.
- Similarly, the altitude difference using the forecast NWM with respect to GNSS altitude is given as:
  (19) $$\epsilon_{fc}=h_{gps}-h_{fc}+\delta_{fc}$$
  where $\delta_{fc}$ is the error resulting from the spatial and diurnal interpolation of the re-analysis of NWM data.
- Finally, the residual ($r_{h}$) between the forecast ($\epsilon_{fc}$) and re-analysis ($\epsilon_{an}$) is computed as:
  (20) $$r_{h}=\epsilon_{fc}-\epsilon_{an}+\delta_{h}$$
  where, $\delta_{h}=\sqrt{\delta_{an}^{2}+\delta_{fc}^{2}}$.

### 2.5. Flight Data

**Figure 9:**
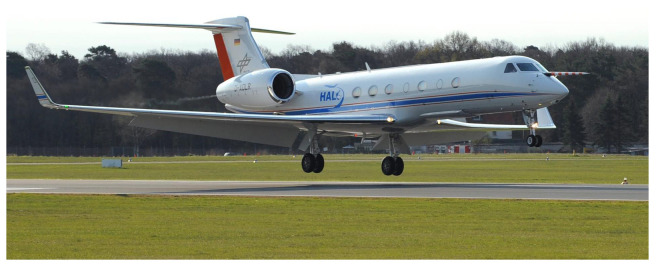
The barometric and GNSS measurements used to validate the BiG-C methodology stem from the DLR HALO research aircraft.

The results of this paper are backed by data collected using the German atmospheric research aircraft (shown in Figure 9) High Altitude and Long Range Research Aircraft (HALO), registration D-ADLR, of the German Aerospace Center (DLR), during the atmospheric research mission “Oxidation Mechanism Observation (OMO)” which was performed in 2015 over parts of Europe and Asia [16]. HALO is based on a Gulfstream G550 business jet. Its combination of long range of more than 8000 km, maximum flight altitude (up to 15 km) [17] payload and flexibility makes HALO a worldwide unique research aircraft.

**Figure 10:**
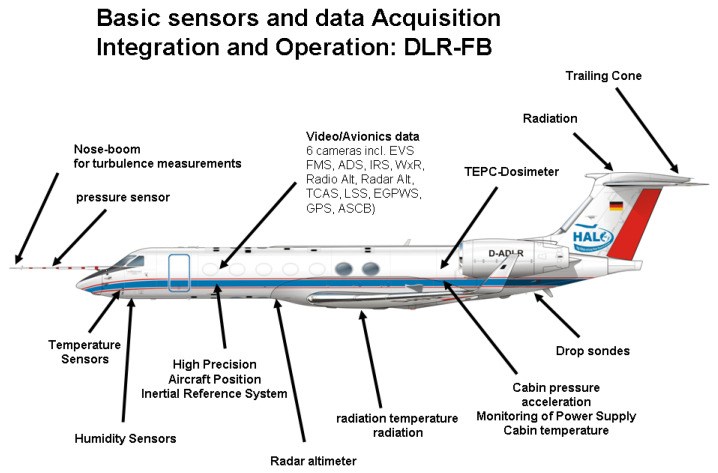
The HALO comprises of several high precision avionics sensors to measure the aircraft position and other air data measurements that is used to validate the BiG-C methodology stems from the DLR HALO research aircraft.

HALO is equipped with a large set of instruments including the Basic HALO Measurement and Sensor System (BAHAMAS) which is used to measure and collect meteorological and aircraft state parameters including static pressure, temperature, and wind data, among several other atmospheric parameters, [17,18]. As shown in Figure 10, the basis sensor system comprises:

- Access to the basic aircraft position data.
- Measurement of the 3-D wind field and turbulence.
- Measurement of pressure, temperature, and humidity with different sensors up to the tropopause level.
- Radiation sensors.
- Surrounding conditions in the aircraft.
- Aircraft Parameter from the Air Data Computer.

The data relevant to this study consist of a track of GNSS position fixes (latitude, longitude, and altitude) along with a measurement of static pressure, temperature, and humidity from the BAHAMAS instrument. The unique ability of the HALO aircraft to collect measurements encompassing a wide range of aircraft altitudes ranging up to 15.5448 km (51,000 feet) above the surface of the earth, makes the data collected using HALO aircraft a perfect candidate to test the validity of the methodology proposed in this paper over a variety of atmospheric conditions. The full details of the OMO mission are described in [16,19].

**Figure 11:**
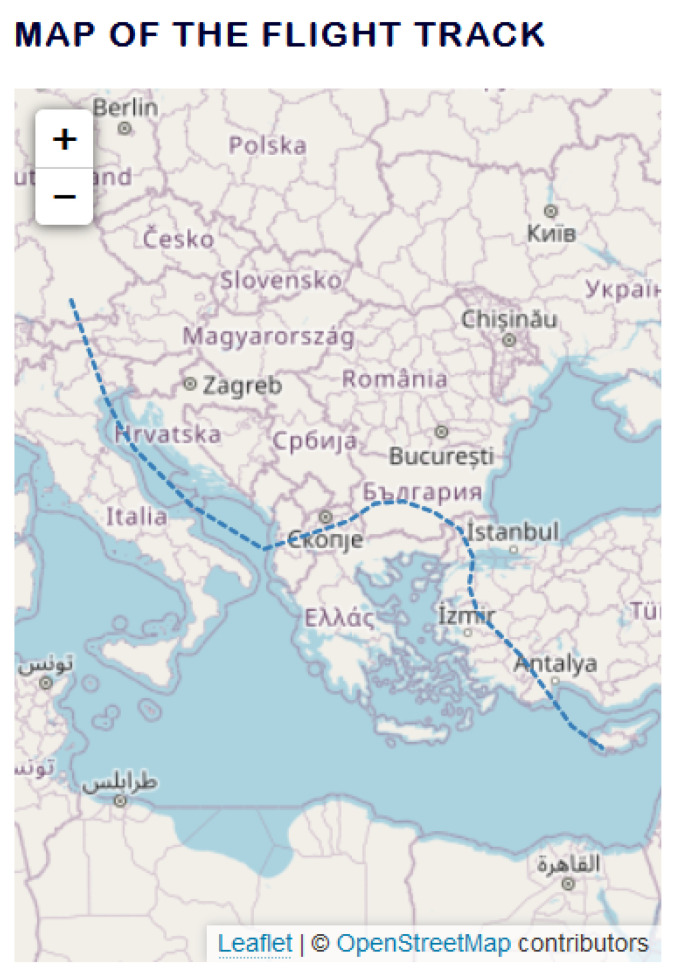
Flight track of one of the flight segments of the OMO mission using the HALO aircraft used for analysis in this study.

The test flights offer a chance to verify the impact of providing accurate reference atmospheric values for barometric altitude measurements in civil aviation. In Figure 11, we show the ground track of the flight data where the test flight took place on 21 July 2015, with take-off from the Oberpfaffenhofen (EDMO), Germany at 09:01 UTC and landing at 12:38 UTC at Paphos (LCPH), Cyprus. The flight data from the OMO mission provides a wide variety of GNSS altitude measurements ranging from ∼15.24 m (50 feet) to about 14.3256 km (47,000 feet), comprising about 12,407 data points, thereby providing an avenue to validate the accuracy of the proposed methodology to reliably estimate the aircraft altitude using the barometric measurements for a wide range of aircraft altitudes.

## 3. Results

**Figure 12:**
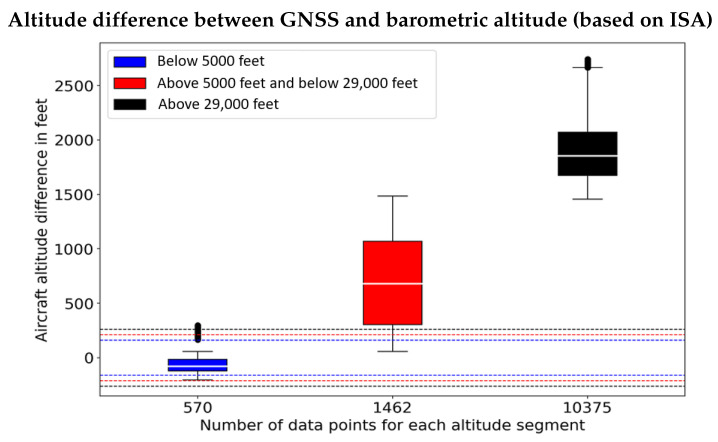
Estimating aircraft altitude using the ISA reference setting (pressure: 1013.25 hPa, temperature: 288.15 K) within the barometric altimeter leads to significant error in comparison to GNSS altitude measurements. The vertical thresholds indicate the VPPL limits, which are the maximum values that the altitude data from the aircraft must satisfy to be eligible for use in providing accurate vertical navigation.

Utilizing the ISA for the reference atmospheric values (pressure: 1013.25 hPa, temperature: 288.15 K) results in vertical errors larger than the required Vertical Path Performance Limit (VPPL) limits; in fact, at greater altitudes, the ISA introduces biases that are greater than two standard deviations. Figure 12 shows the altitude difference between the barometric altitude derived using the ISA reference values and the geometric altitude as measured with GNSS. Note that, the whiskers in Figure 12 are set to indicate 99.7% quantiles as specified in the DO-236C [2]/ED-75D [3]. The deviations resulting from using the ISA as the reference setting within the barometer results in altitude error (in the order of several hundred feet) exceeding the VPPL limits irrespective of aircraft altitude.

The fact that residuals are relatively lower at lower altitudes in comparison to higher aircraft altitudes is a direct consequence of the ISA, which is designed for simplicity, rather than accuracy. From the barometric equation, we know that the altitude is derived by projecting the reference pressure and temperature values to the point of the static pressure measurement by the aircraft. Thus, any deviations in the reference values from the existing pressure and temperature profiles will lead to errors, which grow with increasing altitude.

**Figure 13:**
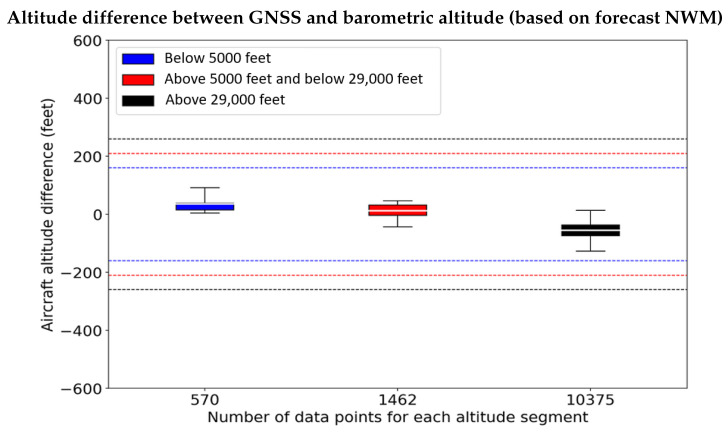
Estimating aircraft altitude using the reference meteorological parameters derived using the forecast NWM within the barometric altimeter in comparison to GNSS altitude measurements leads to errors (Equation (19)) well contained within the VPPL limits regardless of aircraft altitude.

The residual error when using the reference pressure and temperature profiles from the forecast NWM is significantly smaller than those using the ISA. Figure 13 shows the altitude difference (Equation (19)) between the barometric altitude derived using the forecast NWM reference values and the GNSS altitude. From the figure, we can observe that the deviations resulting from using the parameters derived using the proposed methodology in this paper from the forecast NWM, as the reference setting within the barometer causes altitude error well within the VPPL limits for all aircraft altitudes. Comparing the residual of the improved barometric altitudes (Figure 13) with the residual from using the existing methodology based on the ISA reference values (Figure 12), illustrates that at altitudes above 5000 feet, the proposed methodology has residual altitude error significantly smaller by several orders of magnitude in comparison to that achieved with the existing ISA based approach.

The proposed methodology enables estimating the barometric altitude of the aircraft with sufficient accuracy to comply with vertical RNP, as defined in DO-236C [2]/ED-75D [3]. Ensuring vertical repeatability of the altitude measurement systems is crucial as it indicates the ability to estimate aircraft altitude within the desired accuracy uniformly for all phases of aircraft operations. The plot in Figure 13 illustrates this claim, as the residual vertical errors (Equation (20)) are entirely contained by the error budget defined by the VPPL, for all phases of flight.

The altitude residuals ($r_{h}$ shown in Equation (20)) using the reference pressure and temperature from the forecast NWM is in the order of ±12.192 m (40 feet) compared to that determined from the more accurate re-analysis NWM. Figure 13 shows the residual between the altitude difference ($h-h_{gps}$) computed by the forecast and re-analysis of NWM. From the figure, we observe that the forecast NWM provides accurate reference atmospheric profiles enabling accurate estimation of barometric altitude with the unmodeled error within ±12.192 m (40 feet) even at higher altitudes, which is significantly smaller compared to the residual error (Figure 12) obtained when utilizing the ISA.

**Figure 14:**
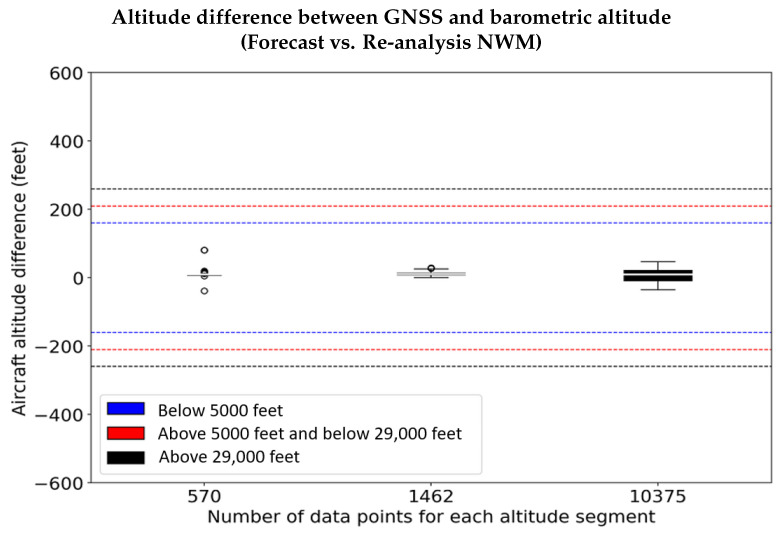
The ERA-5 forecast NWM effectively predicts the reference pressure and temperature, with negligibly small residual altitude difference (Equation (20)) in comparison to accurate re-analysis NWM.

The residual error ($r_{h}$ shown in Equation (20)) has two components: one from the mismatch between the forecast and re-analysis derived barometric altitudes, and the other arising from the difference of the barometric altitudes (estimated using forecast: Equation (19) and re-analysis: Equation (18) NWM, respectively) with the GPS altitude. As shown in Figure 14, at altitudes above 8.8392 km (29,000 feet), the residual error is in the order of 12.192 to 18.288 m (40 to 60 feet), which results primarily due to the deviation of the atmospheric profile predicted by the forecast NWM from the actual state of the atmosphere (as described by the re-analysis NWM). In addition, since the spatial and temporal resolution of the forecast NWM data is limited to 0.125in latitude and longitude and 1 h in time, a part of this residual error also results from the spatial and diurnal interpolation (as described in the Section 2.3) of the meteorological parameters to estimate the reference pressure and temperature matching the exact coordinates of the aircraft and the time of observation.

**Figure 15:**
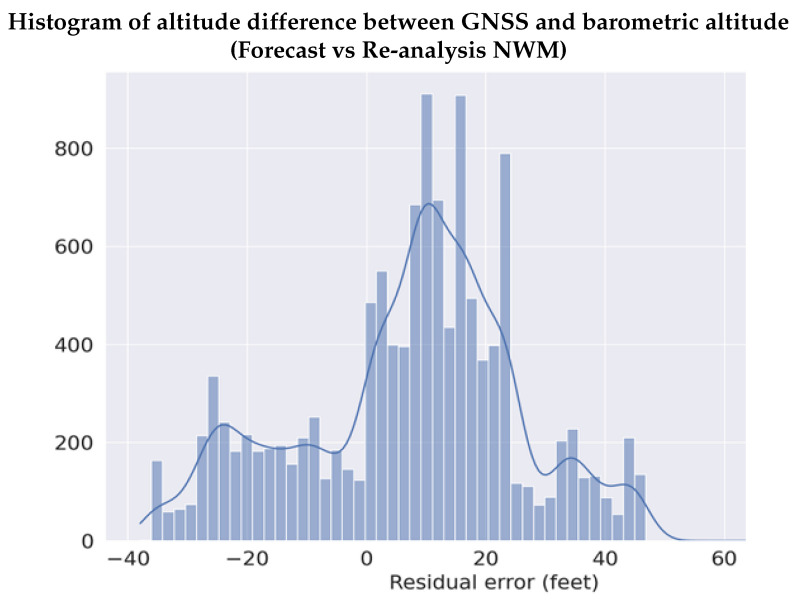
The residual altitude error when using the forecast NWM in comparison to the re-analysis NWM is significantly smaller than the ISA-based method, and, it is completely contained within −12.192 and +12.192 m (−40 and +40 feet), and therefore can be overbounded with a Gaussian distribution.

**Figure 16:**
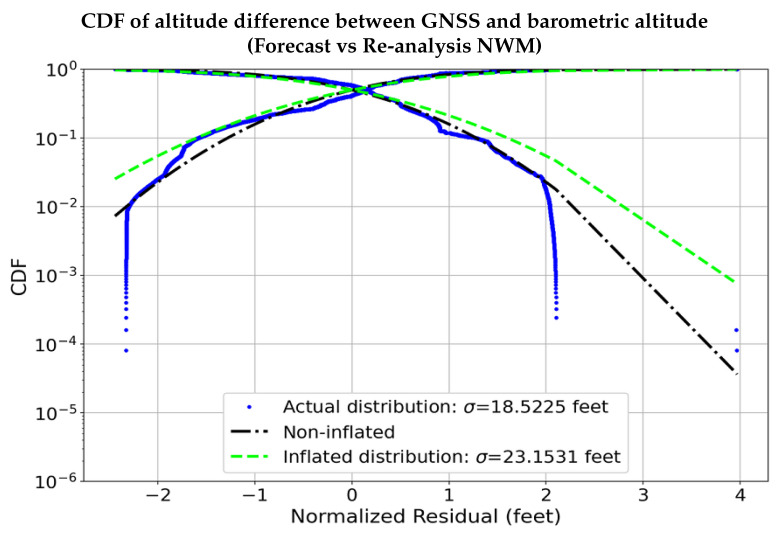
The residual altitude error of the proposed methodology when using the forecast NWM in comparison to the more accurate re-analysis NWM can be overbound with a Gaussian distribution with a standard deviation significantly smaller in comparison to the ISA-derived values.

The unmodeled altitude error ($r_{h}$) is relatively smaller than those derived using re-analysis NWM and can be bound with a zero mean Gaussian distribution. As seen in Figure 15, the distribution of the residual error ($r_{h}$) using the forecast NWM to estimate the barometric altitude in comparison to those derived using re-analysis NWM is completely contained within ±12.192 m (40 feet). Figure 16 shows the CDF of the residuals ($r_{h}$), along with the non-inflated Gaussian distribution, and the overbounding Gaussian distribution. From Figure 16, we notice that the residual error has a standard deviation of about 5.7912 m (19 feet), which is overbound completely with an inflation factor corresponding to 3-sigma, using a zero mean Gaussian distribution resulting in a standard deviation of around ∼7.0104 m (23 feet), which is significantly smaller than the residual error from using the ISA (Figure 12). This clearly illustrated the potential of the proposed methodology to enable accurate vertical navigation using barometric measurements.

## 4. Impact of Improved Vertical Navigation with Barometric Measurements

Satellite-based estimates of altitude are relevant in precision approach and landing applications, where aircraft can execute the “Autoland” function. Here, the key point is that satellite-based estimates of altitude are essentially unbiased, making them sufficiently accurate for such a critical maneuver.

Barometric measurements, on the other hand, are commonly used in en-route applications, where aircraft are at greater altitudes and accuracy is less of a concern. Instead, aircraft rely on so-called flight levels, which are isobaric surfaces, defined in terms of barometric pressures, but labeled in units of altitude for historic reasons. Flight levels serve to deconflict air traffic, as biases in barometric estimates of altitude are common across altitudes: while the actual altitude of a flight level may vary day-to-day or even during one day, the difference in altitude between two flight levels stays fairly constant. As such, it is very common to enforce a minimum vertical separation of 1000 ft between aircraft flying in different directions.

Currently, an aircraft, in an en-route operation, with a barometric malfunction can disrupt air traffic by requiring vertical buffers for vertical separation. Conversely, an aircraft with a GNSS malfunction will be able to better estimate its own altitude, thereby enhancing the performance of its non-GNSS navigation.

The state of the practice for converting between air pressure and altitude is based on the ISA, which cannot account for real-time variability of the atmosphere. As discussed earlier, this model has some advantages, mainly its simplicity in estimating the barometric altitude. Like any model-based approach, it also has shortcomings, but they can be addressed with a complexity that is now accessible to users in civil aviation. With improving weather forecasts and more adequate digital aeronautical communication systems, it seems reasonable that BiG-C will be able to compensate for many of the inherent biases of the ISA, as well as reduce the associated uncertainty. Detailed analysis of the operational aspects of transmitting and subsequent assimilation of real-time weather forecast data into the aircraft is a subject for future work.

An unbiased transformation between geometric altitude and barometric pressure will provide redundancy between barometric altimetry and satellite-based estimation of altitude. Each type of estimate has its own applications, reasons for being, and, more generally, pros and cons. It is also true that currently no effective cross-checks are enabled, because of the erratic nature of the drifting biases in barometric estimates, which BIG-C compensates.

Another compelling prospect is that of supporting vertical navigation performance which can enhance RNP operations. Improvements in the vertical performance for flights within RNP operations are key enablers for advanced concepts such as the systemization of controlled airspace. A Vertical RNP function will reduce the vertical allocation of airspace required for a flight within a systemized operation, thus potentially reducing vertical separation requirements while increasing capacity and flexibility within the airspace. As the systemization of airspace evolves, aircraft will follow routings with a high degree of predictability and certainty from an ATC perspective, allowing for a reduced need for intervention from controllers. The result will be a significantly reduced workload for ATC. In summary, the progressive systemization of airspace, and all contributing functions, will enable improvements in the safety, capacity, flexibility, and efficiency of airspace, especially within volumes with higher complexity.

It is worth highlighting that twinned with the ability to potentially support reduced vertical separation, a key benefit of aligning barometric and geometric altitude indications is the harmonization of operations. The ability of the aircraft to accurately follow a specific geometric profile, especially while pursuing continuous descent and climb operations without needing to change the baseline being used for the calculation of vertical position is of great interest to ATC. This can reduce a great deal of uncertainty, for example, if terrain or obstacles are involved.

Finally, there is the potential for referencing all pressure-based altitudes to the same level, removing the need for a so-called Transition Altitude. The concept of “Transition Altitude” was established to provide a reference altitude level assisting a shift or the transition of the reference setting in the barometric altimeter on board the aircraft from ISA standard values to the locally-referenced values of pressure and temperature usually obtained from the approaching airport. When approaching an airfield, however, it is practical to reference pressure readings to the altitude of the local airfield. This transition is currently executed manually, in the cockpit, and is known to be a potential source of operator error [20]. Removing the transition altitude would, therefore, represent a gain in safety, alongside any of the aforementioned operational benefits.

The proposed methodology behind BIG-C provides a more universal approach to convert barometric measurements to geometric altitude, regardless of aircraft location. This kind of redundancy can improve the reliability of aircraft systems at relatively little expense and high scalability. There are also ways in which these conversion functions reduce the need for pilot input at what is called the “Transition Altitude”, where aircraft go from barometric vertical guidance to geometric vertical guidance (either from GNSS or other navigation sources).

The principle of operation proposed in this paper could be potentially applied to other domains, beyond aviation, for example in UAS (unmanned aerial systems) and UAM (urban air mobility).

## 5. Conclusions

Aircraft vertical guidance is at present primarily dependent on GNSS, and in the event of loss of GNSS, it may lead to inefficient use of airspace and potentially lead to service degradation. Developing a robust altitude estimation approach using barometric measurements provides an alternate means to enable improved vertical guidance to ensure efficient use of airspace both in the presence or absence of GNSS.

Established methods as described in the DO-236C [2]/ED-75D [3] aviation standard, use the ISA values to estimate aircraft altitude using barometric measurements. During the approach of an aircraft to an airport, DO-236C/ED-75D provides a temperature compensation function to correct for any deviations in the reference setting of pressure and temperature used in the barometric altimeter. Moreover, at higher altitudes, the existing approach leads to significant deviations from the GNSS altitude. This conventional approach may lead to availability issues in case of loss of GNSS. This creates a need to develop a new methodology to improve the barometric altitude estimates.

The methodology proposed in this paper provides a means to improve the vertical repeatability of the altitude estimates derived using barometric measurements, regardless of aircraft altitude. BiG-C achieves this by utilizing the forecast NWM data to derive near real-time estimates of reference pressure and temperature profiles considering any anomalous weather conditions such as weather fronts and ducting between the aircraft and the surface of the earth.

BiG-C can achieve altitude estimates accurate to 30 m (100 ft), which is well within the VPPL limits defined in DO-236C/ED-75D, irrespective of the aircraft altitude measurements (ranging from ∼50 to ∼47,000 ft) analyzed from the flight experiment data. In addition, we validated the forecast NWM with respect to the more accurate re-analysis of NWM and showed that forecast NWM provides accurate reference atmospheric profiles enabling unbiased and robust transformation between barometric and geometric altitudes with the unmodeled error in the order of ±12 m (40 ft). Finally, we can completely overbound the residual errors arising from the difference between the altitude estimates using forecast and re-analysis data with a Gaussian distribution with a standard deviation of around ∼7 m (23 ft), which is significantly smaller compared to the residual error obtained utilizing the ISA.

The proposed methodology of improving the altitude estimates using barometric measurements enables a new type of service by applying a more complex conversion than ISA, demonstrating that the method provides a viable solution for vertical RNP based on barometer altitude.

## Figures and Tables

**Table 1:** VPPL specifies the maximum tolerable altitude error of the altitude measurement system for various phases of flight. Adapted with premission from Table 2-1 in RTCA DO-236C ©RTCA/ED-75D ©EUROCAE. Used with permission. All rights reserved [2,3].

Altitude Level (MSL)	Vertical Path Performance Limit (VPPL) (±feet)
At or Below 5000 feet	160
Above 5000 feet to 10,000 feet	210
Above 10,000 feet to 29,000 feet	210
Above 29,000 feet	260

## Data Availability

Not applicable.

## Abbreviations

The following abbreviations are used in this manuscript:

Big-C	Barometric to Geometric Converter
GNSS	Multidisciplinary Digital Publishing Institute
ECMWF	Directory of open access journals
NWM	Numerical Weather Model
ERA	ECMWF re-analysis
ATC	Air Traffic Control
ATM	Air Traffic Management
VNAV	Vertical Navigation
ICAO	International Civil Aviation Organization
ISA	International Standard Atmosphere
PBN	Performance Based Navigation
MSL	Mean-Sea Level
HALO	High Altitude and Long Range Research Aircraft
OMO	Oxidation Mechanism Observation
DLR	German Aerospace Center
BAHAMAS	Basic HALO Measurement and Sensor System
VPPL	Vertical Path Performance Limit
FMS	Flight Management System
LDACS	L-Band Digital Aeronautical and Communication Systems
DME	Distance Measuring Equipment
VOR	VHF Omni-Directional Radar
UAM	Urban Air Mobility
UAS	Unmanned Aerial System
